# Identification of a DYRK1A Inhibitor that Induces Degradation of the Target Kinase using Co-chaperone CDC37 fused with Luciferase nanoKAZ

**DOI:** 10.1038/srep12728

**Published:** 2015-08-03

**Authors:** Rie Sonamoto, Isao Kii, Yuka Koike, Yuto Sumida, Tomoe Kato-Sumida, Yukiko Okuno, Takamitsu Hosoya, Masatoshi Hagiwara

**Affiliations:** 1 Department of Anatomy and Developmental Biology, Graduate School of Medicine, Kyoto University, Yoshida-Konoe-cho, Sakyo-ku, Kyoto 606-8501, Japan; 2 Laboratory of Functional Biology, Graduate School of Biostudies, Kyoto University, Yoshida-Konoe-cho, Sakyo-ku, Kyoto 606-8501, Japan; 3 Laboratory of Chemical Bioscience, Institute of Biomaterials and Bioengineering, Tokyo Medical and Dental University, 2-3-10 Kanda-Surugadai, Chiyoda-ku, Tokyo 101-0062, Japan; 4 Medical Research Support Center, Graduate School of Medicine, Kyoto University, Yoshida-Konoe-cho, Sakyo-ku, Kyoto 606-8501, Japan

## Abstract

The protein kinase family includes attractive targets for drug development. Methods for screening of kinase inhibitors remain largely limited to *in vitro* catalytic assays. It has been shown that ATP-competitive inhibitors antagonize interaction between the target kinase and kinase-specific co-chaperone CDC37 in living cells. Here we show a cell-based method to screen kinase inhibitors using fusion protein of CDC37 with a mutated catalytic 19-kDa component of *Oplophorus* luciferase, nanoKAZ (CDC37-nanoKAZ). A dual-specificity kinase DYRK1A, an importance of which has been highlighted in Alzheimer’s disease, was targeted in this study. We established 293T cells stably expressing CDC37-nanoKAZ, and analyzed interaction between CDC37-nanoKAZ and DYRK1A. We revealed that DYRK1A interacted with CDC37-nanoKAZ. Importantly, point mutations that affect autophosphorylation strengthened the interaction, thus improving signal/noise ratio of the interaction relative to non-specific binding of CDC37-nanoKAZ. This high signal/noise ratio enabled screening of chemical library that resulted in identification of a potent inhibitor of DYRK1A, named CaNDY. CaNDY induced selective degradation of DYRK1A, and inhibited catalytic activity of recombinant DYRK1A with IC_50_ value of 7.9 nM by competing with ATP. This method based on a mutant target kinase and a bioluminescence-eliciting co-chaperone CDC37 could be applicable to evaluation and development of inhibitors targeting other kinases.

Dysregulation of protein kinase activity is implicated in many pathological conditions, which makes protein kinases attractive targets for drug development. Dual-specificity tyrosine-phosphorylation-regulated kinase 1A (DYRK1A), the importance of which has been highlighted by its proposed relationship with early-onset Alzheimer’s disease^1^,^2^,^3^, is a potential target for drug development^4^. In a previous study, we developed a synthetic small molecule, INDY, that potently suppressed the kinase activity of DYRK1A in an *in vitro* kinase assay using recombinant DYRK1A protein^5^.

Kinase-specific co-chaperone CDC37 binds to heat shock protein 90 (HSP90) and client proteins simultaneously, facilitating their interaction^6^,^7^,^8^,^9^. Taipale *et al.* developed a quantitative high-throughput assay to assess interaction between these chaperones and client protein kinases using CDC37 and HSP90β fused with *Renilla* luciferase^10^, and demonstrated a strong correlation between CDC37::kinase and HSP90β::kinase interactions. Stabilization of kinase domain of ABL, SRC, and EGF receptor (EGFR) by inhibitors decreased the HSP90β interaction in living cells^10^,^11^. Polier *et al.* showed that ATP-competitive inhibitors of B-RAF, ErbB2 and EGFR^G719S^ directly antagonize the CDC37 interaction with target kinases *in vitro*^12^. Disruption of HSP90β association and degradation of c-Kit and ErbB2 kinases also occurs after treatment with kinase inhibitors^13^,^14^. Collectively, these studies suggest that the CDC37/HSP90 interaction with client kinases is sensitive to inhibitors targeting the kinases. Therefore, the CDC37/HSP90 interaction assay could be available for screening of kinase inhibitors, instead of conventional *in vitro* kinase assay.

In this study, we developed a cell-based method to screen inhibitors of DYRK1A using fusion protein of CDC37 with a mutated catalytic 19-kDa component of *Oplophorus* luciferase, nanoKAZ (CDC37-nanoKAZ), by modifying the previously reported system^10^,^11^. Using this assay, we revealed that DYRK1A interacted with this chaperone. Furthermore, we found that mutations that affected catalytic activity of DYRK1A enhanced the CDC37 interaction with DYRK1A, which improved signal/noise ratio of the interaction relative to non-specific binding of CDC37-nanoKAZ, and enabled screening of chemical library. Using this system, we examined an original synthetic chemical library, and found a small molecule that acts as an antagonist of the CDC37 interaction with DYRK1A.

## Results

### Treatment with a HSP90 inhibitor decreased the level of DYRK1A protein

**Figure 1:**
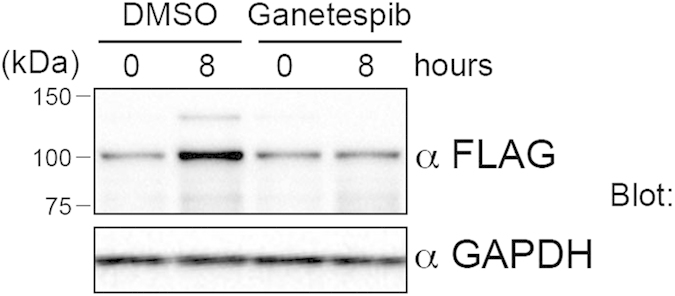
Ganetespib, a HSP90 inhibitor, decreases the DYRK1A protein level. 293T cells were transiently transfected with an expression vector for 3xFLAG-DYRK1A. At 24 h after transfection, the cells were treated with ganetespib (100 nM) and collected 0 and 8 h after treatment. Total cell lysates were subjected to SDS-PAGE followed by Western blot analysis using antibodies against FLAG and GAPDH. In the control group (DMSO), expression of 3xFLAG-DYRK1A increased at 8 h compared to 0 h, and ganetespib suppressed this increase of 3xFLAG-DYRK1A.

To investigate whether DYRK1A is a client kinase of the CDC37/HSP90 system, we used a HSP90 inhibitor, ganetespib. 293T cells were transiently transfected with an expression vector of 3xFLAG-tagged DYRK1A (3xFLAG-DYRK1A). At 24 h after transfection, the cells were treated with ganetespib for the indicated time (0–8 h). Total cell lysates were collected and subjected to SDS-PAGE followed by Western blot analysis. Ganetespib decreased the DYRK1A level compared with the DMSO control (Fig. 1), indicating that stabilization of DYRK1A requires HSP90 activity. This result suggests that DYRK1A is a CDC37/HSP90 client kinase.

### Development of 293T cells expressing CDC37-nanoKAZ

**Figure 2:**
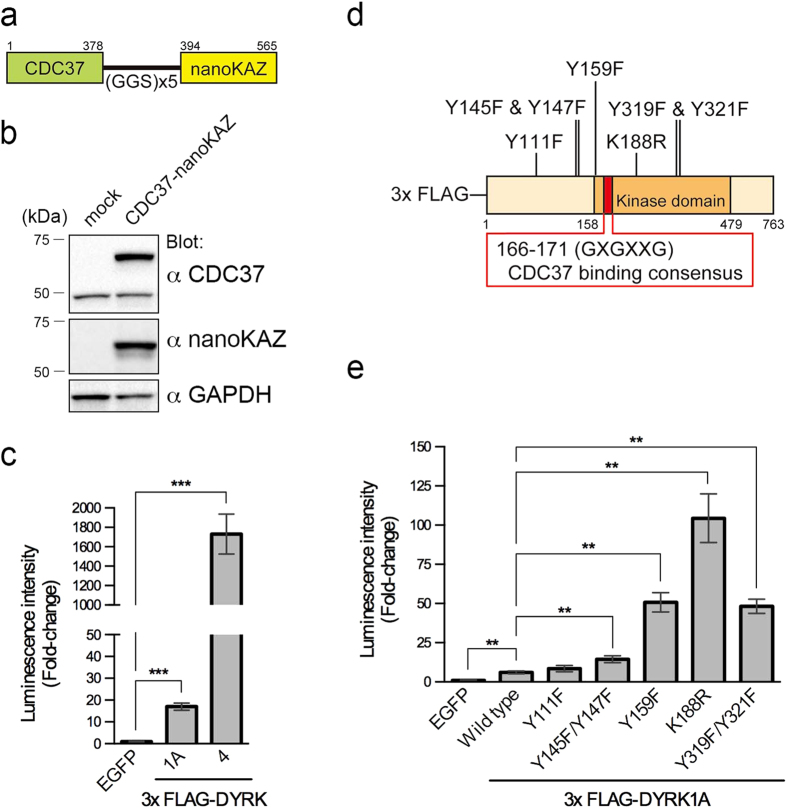
Interaction of DYRK1A with CDC37-nanoKAZ. (**a**) Structure of the CDC37-nanoKAZ protein. CDC37 and nanoKAZ is fused in-frame with a glycine-serine linker. (**b**) Expression of CDC37-nanoKAZ protein in transfected 293T cells. CDC37-nanoKAZ was detected with antibodies against CDC37 or nanoKAZ. GAPDH was detected as an internal control. (**c**) Interactions of 3xFLAG-DYRK1A and 3xFLAG-DYRK4 with CDC37-nanoKAZ. Luminescence intensities of CDC37-nanoKAZ associated with 3xFLAG-DYRK1A and 3xFLAG-DYRK4 proteins, which were bound on 96-well plates coated with antibody against FLAG, are shown as fold-changes relative to that with EGFP (luminescence due to non-specific binding of CDC37-nanoKAZ). Bar graphs show means ± SD, ***p < 0.001 (n = 8). (**d**) Cartoon illustrating the structure of 3xFLAG-DYRK1A, in which the mutations used in this study and the CDC37 consensus binding site are shown. (**e**) Interactions of intact and mutated DYRK1A proteins with CDC37-nanoKAZ. Luminescence intensities of CDC37-nanoKAZ associated with 3xFLAG-DYRK1A proteins are shown as fold-changes relative to that with EGFP. Bar graphs show means ± SD, **p < 0.01 (n = 5).

To assess the CDC37 interaction with DYRK1A quantitatively, we developed an expression vector of CDC37 fused with nanoKAZ, a mutated catalytic 19-kDa component of *Oplophorus* luciferase^15^,^16^. The structure of CDC37-nanoKAZ is shown in Fig. 2a. Codon-optimized nanoKAZ was fused to the carboxyl-terminus of CDC37, because carboxyl-terminal tagging of CDC37 did not significantly affect its function^10^,^11^. 293T cells were transiently transfected with the CDC37-nanoKAZ vector. At 48 h after transfection, total cell lysates were collected. Endogenous CDC37 was detected in Western blot analysis, along with a slower migrating band for the exogenous CDC37-nanoKAZ fusion protein (Fig. 2b). An antibody against nanoKAZ also recognized CDC37-nanoKAZ (Fig. 2b). The luminescence intensity for CDC37-nanoKAZ in total cell lysate determined using its substrate, *bis*-coelenterazine, had a glow luminescence pattern (Supporting Figure S1a), as previously reported in nanoKAZ^16^. We then established stable 293T cell lines that expressed CDC37-nanoKAZ or nanoKAZ.

### Mutations that affect autophosphorylation of DYRK1A strengthen the interaction with CDC37-nanoKAZ

Although the interaction between DYRK1A and CDC37/HSP90 had not been demonstrated yet, DYRK4 was categorized as a strong client kinase of the CDC37/HSP90 system^10^. To examine whether CDC37-nanoKAZ works as a reporter to detect the CDC37 interaction with its client kinase, we investigated interaction of CDC37-nanoKAZ with DYRK1A and DYRK4. The 293T cells stably expressing CDC37-nanoKAZ were transiently transfected with expression vectors for 3xFLAG-DYRK1A and 3xFLAG-DYRK4 in a 96-well plate. An expression vector for EGFP was also transfected as a negative control. Two days after transfection, the cells were lysed and cleared cell lysates were incubated in a 96-well plate coated with antibody against FLAG peptide. After binding at 4 °C for 3 h followed by extensive wash, *bis*-coelenterazine was injected into the wells and the luminescence kinetics was measured. The values for the initial luminescence intensity (*I*_max_) were processed statistically. The amounts of 3xFLAG-DYRK1A and 3xFLAG-DYRK4 bound on the antibody-coated well were quantified with a horseradish peroxidase (HRP) conjugated antibody against FLAG. The levels of bound proteins were almost the same (within ± 2.0%) between samples (Supporting Figure S1b).

Significant increases of the luminescence signal in the transfection of either 3xFLAG-DYRK1A or 3xFLAG-DYRK4 with CDC37-nanoKAZ were detected (Fig. 2c), compared with EGFP (luminescence caused by non-specific binding of CDC37-nanoKAZ), indicating that CDC37-nanoKAZ functions as luminescence reporter to detect the CDC37 interaction with DYRK family kinases. 3xFLAG-DYRK4 interacted with CDC37-nanoKAZ 102-fold more strongly than 3xFLAG-DYRK1A (Fig. 2c), suggesting that DYRK1A is a weak client kinase compared with DYRK4. We next examined the interaction of CDC37-nanoKAZ with DYRK1A harboring substitution mutations. The mutation sites are shown in Fig. 2d. Interestingly, a 3xFLAG-tagged kinase-dead mutant of DYRK1A, in which Lys188 was substituted to Arg (K188R), interacted with CDC37-nanoKAZ 17-fold more strongly than intact DYRK1A (Fig. 2e), whereas the amounts of bound proteins were similar (Supporting Figure S1c). Lys188 is essentially involved in orienting ATP for catalysis^17^, and thus the K188R mutant lost its kinase activity^17^. The strengthened interaction of the K188R mutant suggests that the kinase activity is involved in the CDC37 interaction.

To further confirm whether the kinase activity is involved in the CDC37 interaction, an expression vector for 3xFLAG-DYRK1A harboring two mutations in the activation loop was prepared, in which Tyr319 and Tyr321 were substituted to Phe (Y319F/Y321F). The requirement for phosphorylation of these tyrosine residues remains uncertain^17^,^18^,^19^,^20^, but the Y319F/Y321F mutant lost its kinase activity^17^,^21^,^22^. 3xFLAG-DYRK1A (Y319F/Y321F) interacted with CDC37-nanoKAZ 8.0-fold more strongly than intact DYRK1A (Fig. 2e). These results suggest that inactive forms of DYRK1A interact with CDC37 more strongly than the active form.

The kinase-dead mutants (K188R and Y319F/Y321F) were unable to catalyze autophosphorylation, in addition to substrate phosphorylation. If autophosphorylation affects the CDC37 interaction, the autophosphorylation sites around the CDC37-interacting glycine-rich loop would be involved in the interaction (see Fig. 2d). Therefore, we prepared expression vectors of 3xFLAG-DYRK1A harboring mutations at Tyr111 (Y111F), Tyr145 and Tyr147 (Y145F/Y147F), and Tyr159 (Y159F), which are the reported autophosphorylation sites^17^,^20^, and examined the interaction with CDC37-nanoKAZ. 3xFLAG-DYRK1A (Y159F) interacted with CDC37-nanoKAZ 8.4-fold more strongly than intact DYRK1A (Fig. 2e). The other mutants, Y111F and Y145F/Y147F, interacted with CDC37-nanoKAZ 1.4- and 2.4-fold more strongly than intact DYRK1A, respectively (Fig. 2e). The amounts of bound these mutant proteins were similar to that of wild-type (Supporting Figure S1c). Furthermore, the intact and mutated DYRK1A proteins did not interact with nanoKAZ (Supporting Figure S1d). These results indicate a possibility that the interaction of DYRK1A with CDC37 is regulated by autophosphorylation.

### DYRK1A interacts with HSP90β fused with nanoKAZ

**Figure 3:**
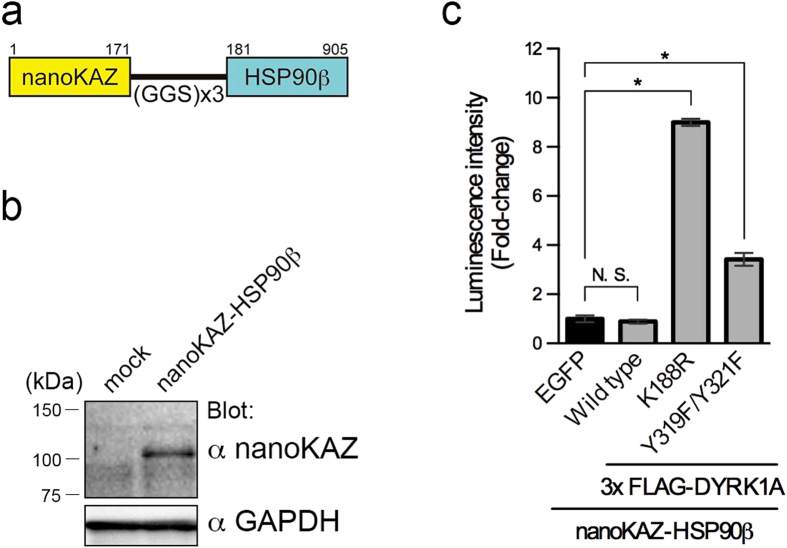
Interaction of DYRK1A with nanoKAZ-HSP90β. (**a**) Structure of the nanoKAZ-HSP90β protein. (**b**) Expression of nanoKAZ-HSP90β protein in transfected 293T cells. nanoKAZ-HSP90β was detected with antibodies against nanoKAZ. GAPDH was detected as an internal control. (**c**) Interactions of intact and mutated DYRK1A proteins with nanoKAZ-HSP90β. Luminescence intensities of nanoKAZ-HSP90β associated with 3xFLAG-DYRK1A proteins are shown as fold-changes relative to that with EGFP. Bar graphs show means ± SD, *p < 0.05 (n = 4).

CDC37 binds to HSP90 and client kinases simultaneously, and facilitates their interaction^6^,^7^,^8^,^9^. We therefore examined the interaction between DYRK1A and HSP90β. The structure of nanoKAZ-HSP90β is shown in Fig. 3a. Codon-optimized nanoKAZ was fused to the amino-terminus of HSP90β. 293T cell line stably expressing nanoKAZ-HSP90β was established. Expression of nanoKAZ-HSP90β was confirmed by Western blot analysis (Fig. 3b). To measure the interaction, the stable 293T cell lines were transiently transfected with expression vectors for 3xFLAG-DYRK1A and its mutants, and processed as described above. The levels of bound proteins were almost the same (within ± 4.0%) between samples (Supporting Figure S2).

The luminescence signal of nanoKAZ-HSP90β in the 3xFLAG-DYRK1A-bound well was almost the same as EGFP (luminescence caused by non-specific binding of nanoKAZ-HSP90β) (Fig. 3c). On the other hand, significant interactions of nanoKAZ-HSP90β with the DYRK1A mutants were detected compared with EGFP (Fig. 3c). The mutants of K188R and Y319F/Y321F interacted with nanoKAZ-HSP90β 10- and 3.8-fold more strongly than intact DYRK1A, respectively (Fig. 3c). These results demonstrate that CDC37-nanoKAZ functions as the interaction reporter with higher signal/noise ratio than nanoKAZ-HSP90β.

### Interaction between DYRK1A (Y319F/Y321F) and CDC37-nanoKAZ is antagonized by inhibitors of DYRK1A

To examine whether small molecule inhibitors of DYRK1A antagonize the CDC37 interaction with DYRK1A, we used the substituted mutant of 3xFLAG-DYRK1A (Y319F/Y321F) with higher luminescence, but not the K188R mutant because several DYRK1A inhibitors utilize Lys188 in the ATP-binding pocket^5^. The stable cell lines were transfected with the expression vector of 3xFLAG-DYRK1A (Y319F/Y321F), and cultured for two days in a 96-well plate. The cells were then treated with the indicated concentrations of small molecules for one hour. Cell lysates were added to FLAG antibody-coated 96-well plates and luminescence intensities were measured. The amount of bound 3xFLAG-DYRK1A was measured using a HRP-conjugated antibody against FLAG. The relative *I*_max_ for CDC37-nanoKAZ was normalized using the amount of bound protein.

**Figure 4:**
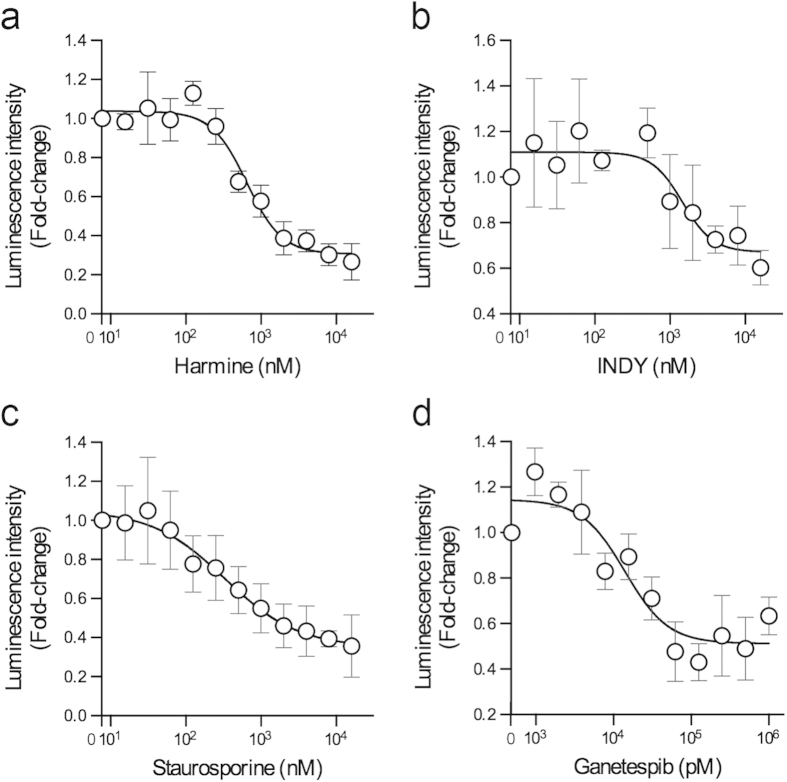
DYRK1A inhibitors and a HSP90 inhibitor antagonize the interaction of CDC37-nanoKAZ with a DYRK1A mutant. (**a-d**) 293T cells stably expressing CDC37-nanoKAZ were transiently transfected with an expression vector for 3xFLAG-DYRK1A (Y319F/Y321F) and then treated with the indicated concentrations of harmine (**a**), INDY (**b**), staurosporine (**c**), and ganetespib (**d**). Luminescence intensities are shown as fold-changes relative to that at 0 nM, normalized to the amount of 3xFLAG-DYRK1A (Y319F/Y321F) bound on a 96-well plate. Points are means ± SD (n = 3). Representative dose-response curves with Hill slopes are shown.

Treatment of the cells with harmine, an inhibitor of DYRK1A^5^,^23^,^24^,^25^,^26^, decreased the relative luminescence intensity with a half maximal effective concentration (EC_50_) of 647 nM (Fig. 4a), indicating that harmine antagonizes the interaction of CDC37-nanoKAZ with 3xFLAG-DYRK1A in living cells. Another DYRK1A inhibitor, INDY^5^, also decreased the relative luminescence intensity with an EC_50_ of 1.5 μM (Fig. 4b), and a broad-spectrum protein kinase inhibitor, staurosporine, decreased the intensity with an EC_50_ of 333 nM (Fig. 4c). In addition to kinase inhibitors, treatment with ganetespib, a HSP90 inhibitor, decreased the intensity with an EC_50_ of 14 nM (Fig. 4d), indicating that HSP90 inhibition also antagonizes the interaction of CDC37-nanoKAZ with 3xFLAG-DYRK1A (Y319F/Y321F). These small molecules did not inhibit the luminescence activity of CDC37-nanoKAZ (Supporting Figure S3). These results demonstrate that a cell-based reporter system based on a DYRK1A mutant and CDC37-nanoKAZ is available for evaluation of the effects of small molecules on the DYRK1A/CDC37 interaction in living cells.

### Evaluation of inhibitors of DYRK1A in a synthetic chemical library

**Figure 5:**
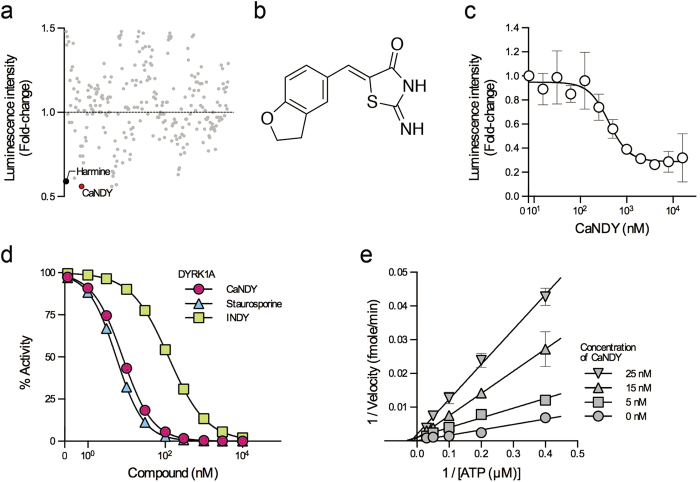
Identification of a potent inhibitor of DYRK1A. (**a**) A total of 253 compounds from our original synthetic chemical library were examined. These compounds were used at 4 μM. Relative luminescence intensities are shown. Harmine was used as a positive control and is indicated by the black point. The red point indicates CaNDY. (**b**) Structure of CaNDY. (**c**) CaNDY antagonized the interaction between DYRK1A and CDC37 complex. 293T cells expressing CDC37-nanoKAZ were transiently transfected with an expression vector for 3xFLAG-DYRK1A (Y319F/Y321F) and then treated with the indicated concentrations of CaNDY. The points represent means ± SD (n = 3). Representative dose-response curves with Hill slopes are shown. (**d**) CaNDY inhibited the catalytic activity of DYRK1A in an *in vitro* kinase assay. Recombinant DYRK1A was incubated with the substrate peptide DYRKtide-F in the presence of the indicated concentrations of small molecules. CaNDY, INDY, and staurosporine inhibited the kinase activity with IC_50_ values of 7.9 nM, 122 nM, and 5.4 nM, respectively. Representative dose-response curves with Hill slopes are shown. (**e**) Double-reciprocal plots showing the competitive inhibition of ATP by CaNDY. DYRK1A kinase activity was measured at the indicated concentrations of CaNDY and ATP. Reciprocal velocity was plotted versus 1/[ATP]. *K*_*m*_ = 14.1 μM and *K*_*i*_ = 1.92 nM.

The bioluminescent reporter system based on CDC37-nanoKAZ was used for evaluation of a library of compounds. Cells expressing CDC37-nanoKAZ and 3xFLAG-DYRK1A (Y319F/Y321F) were treated with small molecules and processed as described above. The relative luminescence intensities are shown in Fig. 5a. The compound that decreased the luminescence intensity to the lowest level was (*Z*)-5-[(2,3-dihydrobenzofuran-5-yl)methylene]-2-iminothiazolidin-4-one (referred to as CaNDY: CDC37 association inhibitor for DYRK1A). The structure of CaNDY is shown in Fig. 5b. Treatment of the cells with CaNDY decreased the luminescence intensity with an EC_50_ of 409 nM (Fig. 5c). CaNDY did not inhibit the luminescence activity of CDC37-nanoKAZ (Supporting Figure S4).

To examine whether CaNDY inhibits the catalytic activity of DYRK1A, we performed an *in vitro* kinase assay using recombinant DYRK1A and a substrate peptide. In this assay, CaNDY inhibited substrate phosphorylation with a half-maximal inhibitory concentration (IC_50_) of 7.9 nM (Fig. 5d), whereas staurosporine and INDY had IC_50_ values of 5.4 and 122 nM, respectively. We also examined whether CaNDY is an ATP-competitive inhibitor. The double-reciprocal plots showed first-order inhibitory kinetics, demonstrating that CaNDY competed with ATP on a single site of DYRK1A (Fig. 5e). These results demonstrate that CaNDY is the ATP-competitive type I inhibitor that antagonizes the CDC37 interaction with DYRK1A.

**Figure 6:**
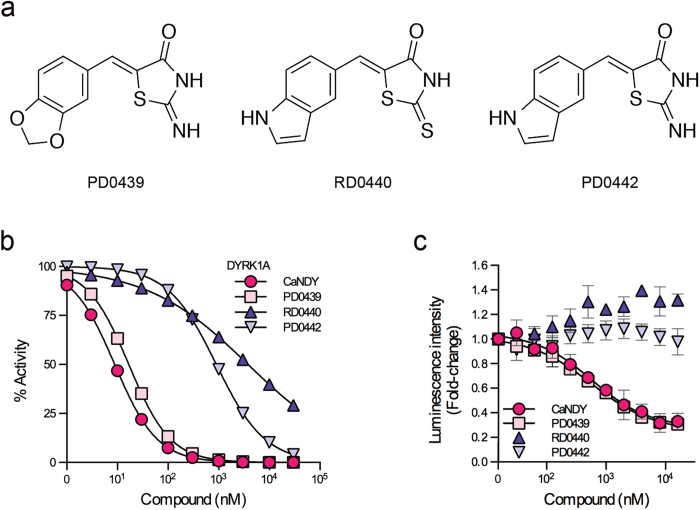
Structural requirement of CaNDY. (**a**) Structures of PD0439, RD0440, and PD0442. (**b**) *In vitro* kinase assay using these small molecules, as performed in Fig. 5d. (**c**) CDC37-nanoKAZ binding assay using these small molecules, as performed in Fig. 5c.

Next we investigated structural requirement for the unique inhibitory effect of CaNDY. We selected three small molecules, PD0439, RD0440, and PD0442, from the library, which are structurally similar to CaNDY (Fig. 6a). PD0439, which the dihydrobenzofuran structure of CaNDY was replaced with methylenedioxybenzene, inhibited the *in vitro* kinase activity of DYRK1A with IC_50_ value of 16.7 nM (Fig. 6b). RD0440 and PD0442 with indole substructure showed weak inhibitory activity against recombinant DYRK1A, compared with CaNDY and PD0439 (Fig. 6b). These results indicate that oxygen atom attached to the benzene and iminothiazolidinone of CaNDY and PD0439 are key pharmacophores for exhibiting strong inhibitory effect. Consistently, PD0439 antagonized the CDC37-nanoKAZ interaction with 3xFLAG-DYRK1A (Y319F/Y321F) with EC_50_ value of 573 nM (Fig. 6c). On the other hand, RD0440 and PD0442 did not antagonize the interaction (Fig. 6c). These results indicate that the interactions detected by CDC37-nanoKAZ reflect the potencies of DYRK1A inhibition *in vitro*, and that weak inhibitors of DYRK1A do not antagonize the CDC37 interaction.

### CaNDY is a potent and selective inhibitor of DYRK family kinases

**Figure 7:**
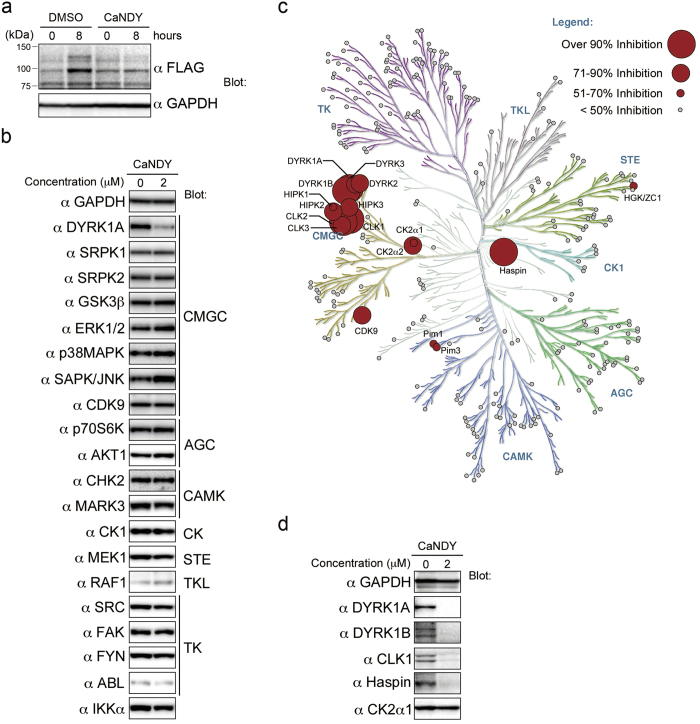
CaNDY induces selective degradation of DYRK1A. (**a**) 293T cells were transiently transfected with an expression vector for 3xFLAG-DYRK1A. At 24 h after transfection, the cells were treated with CaNDY (2 μM) and collected 0 and 8 h after treatment. (**b**) HEK293 cells were cultured in the presence of CaNDY (2 μM) for four days. The indicated kinases and GAPDH were detected by Western blot analysis using antibodies against each protein. The standard classification of these kinases^37^ is shown. (**c**) Map of the inhibitory activities of CaNDY on a kinase dendrogram^37^. Percentage inhibitions by 1 μM CaNDY were measured for a panel of 275 kinases. Red circles indicate inhibited kinases and the circle size indicates the percentage inhibition. Illustration reproduced courtesy of Cell Signaling Technology, Inc. (www.cellsignal.com). (**d**) Protein expression of the kinases inhibited by over 90% in (**c**) and CK2α, which was inhibited by 65%, was detected by Western blot analysis using antibodies against each protein.

We investigated whether CaNDY destabilizes DYRK1A as ganetespib does. In the same experiment as Fig. 1, CaNDY decreased the 3xFLAG-DYRK1A level compared with the DMSO control (Fig. 7a). Next we examined whether CaNDY decreased endogenous DYRK1A protein. HEK293 cells were cultured for 0–4 days with CaNDY (2 μM) and then subjected to SDS-PAGE followed by Western blot analysis using antibodies against DYRK1A and GAPDH. Expression of DYRK1A increased during the 4-days culture, and CaNDY prevented accumulation of endogenous DYRK1A (Fig. 7b and Supporting Figure S5), whereas ganetespib also prevented it (Supporting Figure S5). The treated cells were also subjected to reverse transcription followed by quantitative PCR analysis. Expression of mRNA coding for DYRK1A was slightly decreased by CaNDY (Supporting Figure S6), but this decrease was not significant compared with the reduction of endogenous DYRK1A protein. Treatment with a proteasome inhibitor partially prevented the CaNDY-mediated reduction of 3xFLAG-DYRK1A (Supporting Figure S7), suggesting that proteasome is involved in the degradation of DYRK1A. Furthermore, CaNDY did not decrease pre-accumulated DYRK1A in an experiment using cycloheximide (Supporting Figure S8). These results suggest that CaNDY selectively destabilizes newly synthesized DYRK1A.

To check selectivity of CaNDY for other kinases, we first examined protein expression of several kinases in HEK293 cells treated with CaNDY (2 μM) for 4 days. CaNDY did not decrease the other 19 kinases (Fig. 7b). Second, *in vitro* inhibitory activities of 1 μM CaNDY against a panel of 275 recombinant kinases were assessed. CaNDY inhibited DYRK1A, DYRK1B, CLK1, CLK2, and Haspin by over 90% (Fig. 7c, Supporting Figure S9a, and Supporting Table S1). The *in vitro* IC_50_ of CaNDY for DYRK1B was 24.1 nM, compared with 2.36 nM for staurosporine and 69.1 nM for INDY (Supporting Figure S9b). Third, protein expression of these kinases targeted by CaNDY (DYRK1A, DYRK1B, CLK1, and Haspin) was reassessed by Western blot analysis as performed in Fig. 7b. CaNDY also decreased expression of the target kinases, but did not decrease CK2α, which was inhibited by 65% in the kinase panel assay (Fig. 7d). These results demonstrate that CaNDY is a potent and selective inhibitor of DYRK family kinases.

## Discussion

We developed the bioluminescent reporter assay for evaluation of DYRK1A inhibitors, based on the interaction of CDC37-nanoKAZ with the DYRK1A Y319F/Y321F mutant. Using this assay, we identified a potent inhibitor of DYRK1A in our chemical library. This finding indicates that this reporter assay could contribute to chemical screen as well as the evaluation of pre-existing inhibitors.

This study revealed that DYRK1A is a client kinase of the CDC37/HSP90 system. The interaction site for CDC37 on client kinases was localized to the N-lobe of the catalytic domain 27,28,29,30. Phage display and liquid chromatography-tandem mass spectrometry identified a canonical glycine-rich loop (GXGXXG) in the N-lobe as a CDC37-interacting motif^31^. DYRK1A possesses this conserved loop (^166^GKGSFG^171^) in the N-lobe (Fig. 2d), consistent with our findings.

The requirement of the HSP90 activity in production of DYRK1A suggests that DYRK1A interacts with the CDC37/HSP90 system during its maturation process. Our study revealed that DYRK1A is a weak client kinase. Thus, CDC37 may be released from DYRK1A after its maturation. Autophosphorylation occurs during maturation process^19^,^32^,^33^, indicating a possibility that autophosphorylation of several residues, such as Tyr159, is required for the dissociation step. Interestingly, the results indicate the importance of Tyr159, which is the autophosphorylation site closest to the consensus CDC37 binding site. DYRK1A changes its substrate from a tyrosine to a serine/threonine during the maturation process^19^. In the initial step after synthesis, DYRK1A autophosphorylates its tyrosine residues, and then phosphorylates serine/threonine residues of itself and of substrates^17^,^19^,^21^. Thus, Tyr159 autophosphorylation may occur in the initial step, leading to the hypothesis that CDC37 transiently interacts with the immature tyrosine kinase form of DYRK1A. Chaperones assist folding of newly synthesized proteins and dissociate once the proteins are folded^34^. Several reports suggest that the CDC37 function is specific to immature and inactive forms of kinases^8^,^35^, and CDC37 plays a pivotal role in the kinome by protecting nascent chains from degradation during or immediately after translation^8^. These reports support the possibility described above.

CaNDY and ganetespib affect the complex of DYRK1A and the CDC37/HSP90 chaperone system. The treatment of the cells with ganetespib for 8 h prevented accumulation of the DYRK1A protein, suggesting that the HSP90 activity is necessary to accumulate the DYRK1A protein. It has been demonstrated that CDC37 is required for the interaction between its client kinase and HSP90^6^,^7^,^8^,^9^. Thus, antagonistic effect of CaNDY against the CDC37 interaction probably causes degradation of DYRK1A during its maturation process.

The bioluminescent reporter system has following beneficial points for chemical screening. First, high signal/noise ratio: we used nanoKAZ for our reporter system, which is the mutated 19-kDa component of *Oplophorus* luciferase^16^. Compared with 36-kDa *Renilla* luciferase, this small luciferase nanoKAZ enables us to make fusion proteins without affecting the function of the fusion partner. This may contribute to the high signal/noise ratio of the CDC37-nanoKAZ interaction. In addition, nanoKAZ has strong luminescence. nanoKAZ showed only 1.4-fold strong luminescence compared with *Renilla* luciferase in *in vitro* assay using recombinant proteins^16^; but, nanoKAZ was codon-optimized by preferred human codon-optimized method^15^, which resulted in over 30-fold stronger luminescence intensity of nanoKAZ than that of *Renilla* luciferase in mammalian cells^15^. The strong luminescence permits accurate and reproducible measurements of the CDC37-nanoKAZ interaction. Second, we used kinase-dead mutants in our reporter assay. Transient overexpression of a kinase in a living cell affects the cellular signaling cascades, resulting in excessive proliferation or cell death, whereas transient overexpression of a dead kinase should be less influential to the cells, leading to reliable results. Moreover, our method would contribute to identification of not only type I inhibitor such as CaNDY but also type II inhibitor that targets ATP-binding pocket of inactive form. We note that the bioluminescence screening system can also identify inhibitors of HSP90 and irreversible inhibitors of nanoKAZ as hit compounds. These small molecules should be omitted by appropriate secondary screening.

Using the luminescence reporter system, we identified CaNDY as the ATP-competitive strong inhibitor of DYRK family kinases. In addition to the *in vitro* inhibitory activity, CaNDY destabilized DYRK1A in living cells, which may be due to the antagonistic effect of CaNDY against the interaction between DYRK1A and CDC37. CaNDY decreases DYRK1A molecules in cells, thus would efficiently suppresses the DYRK1A activity, compared with simple inhibition of the kinase activity.

Our results suggest that CDC37/HSP90 interacts with some client kinases only when the clients are immature or inactive. It is unclear how many kinases interact with CDC37/HSP90 when mutated to inactive forms, but our approach should be applicable for almost all kinases that possess the conserved CDC37 binding site. Kinases are the largest subset of druggable targets, with approximately 2% of all genes encoding kinases that regulate cellular signaling pathways involved in a wide range of diseases. Thus, we believe that the method presented here is an innovative tool that could be adapted to other kinases of interest for drug discovery.

## Methods

### Materials

Small molecules (CaNDY, PD0439, RD0440, and PD0442) were prepared as described in the Supporting Information. INDY was prepared as described previously^5^. Ganetespib, harmine, and staurosporine were purchased from MedChem Express (Princeton, NJ, USA), Tokyo Chemical Industry (Tokyo, Japan), and Tocris Bioscience (Bristol, UK), respectively. These compounds were dissolved in DMSO (Hybri-MAX^TM^, Sigma-Aldrich, St. Louis, MO, USA). Puromycin was obtained from Nacalai Tesque (Kyoto, Japan) and dissolved in PBS. *bis*-Coelenterazine (BlueSyn C) was purchased from Synchem (Altenburg, Germany) and dissolved in ethanol, after which the solution was divided into aliquots and lyophilized. Epoxomicin, a proteasome inhibitor, was obtained from Peptide Institute (Osaka, Japan). Cycloheximide was purchased from Sigma-Aldrich.

### Antibodies

A rabbit polyclonal antibody against the 19-kDa component of *Oplophorus* luciferase was kindly provided by Dr. Satoshi Inouye (Yokohama Research Center, JNC Corporation, Yokohama, Japan). Mouse monoclonal anti-FLAG peptide (clone M2) and horseradish peroxidase-conjugated antibodies were purchased from Sigma-Aldrich. Mouse monoclonal antibodies against SRPK1, SRPK2 (23/SRPK2), and CDK9 (D-7) were purchased from Pharmingen (BD Biosciences, San Jose, CA, USA), BD Transduction Laboratories (BD Biosciences), and Santa Cruz Biotechnology (Santa Cruz, CA, USA), respectively. Mouse monoclonal antibody against GAPDH (6C5) and rabbit polyclonal antibodies against CLK1 and Haspin were obtained from Abcam (Cambridge, UK). Rabbit monoclonal antibodies against CDC37 (D11A3), GSK3β (27C10), AKT1 (C73H10), MEK1 (30C8), SRC (32G6) and rabbit polyclonal antibodies against DYRK1A, DYRK1B, p42/p44 MAPK (ERK1/2), p38 MAPK, SAPK/JNK, p70S6K, CHK2, MARK3, CK1, CK2α, RAF1, FAK, FYN, c-ABL, and IKKα were purchased from Cell Signaling Technology (Beverly, MA, USA). These commercially available 1^st^ antibodies and its reference numbers are listed in Table S2. HRP-linked anti-rabbit and anti-mouse IgG were purchased from GE Healthcare Life Sciences (Pittsburgh, PA, USA) and Abcam, respectively.

### Vector construction

A PCR-amplified fragment of the codon-optimized gene for nanoKAZ (GeneBank Accession No. AB823628)^15^ was fused in-frame to the carboxyl terminus of that of the CDC37 gene and to the amino-terminus of that of HSP90β (HSP90AB1 gene product). The fused CDC37-nanoKAZ and nanoKAZ-HSP90AB1 gene fragments were subcloned into a pCAGIPuro vector using an In-Fusion^®^ HD Cloning kit (Clontech, Takara Bio, Shiga, Japan). The nanoKAZ gene was also subcloned into the pCAGIPuro vector. The pCAGIPuro vector, an IRES-based bicistronic expression vector in which the gene of interest and a puromycin resistant gene are expressed from a single mRNA, enables almost all of the cells selected with puromycin to express the gene product.

Expression vectors for DYRKs and DYRK1A mutants were constructed in a pcDNA5/FRT/TO vector (Life Technologies, Thermo Fisher Scientific) (Kii *et al.* manuscript in revision). In brief, PCR-amplified fragments of 3xFLAG-tagged DYRKs were fused in-frame to the amino-terminus of EGFP via the F2A peptide sequence by overlap-extension PCR, and the combined fragments were inserted into the pcDNA5/FRT/TO vector. The EGFP gene was also inserted into the pcDNA5/FRT/TO vector. The reconstituted vector sequences are available upon request.

### Cell culture and transfection

293T and HEK293 cells were maintained in low glucose Dulbecco’s modified Eagle’s medium (Nacalai Tesque) supplemented with 10% fetal bovine serum (Sigma-Aldrich), 100 units/mL penicillin and 100 μg/mL streptomycin (Nacalai Tesque). Cells were transfected with plasmid DNAs using polyethylenimine ‘MAX’ (Polysciences, Warrington, PA, USA) and then selected with Puromycin (Nacalai Tesque) for pCAGIPuro vectors to establish stable cell lines.

### Western blot analysis

Western blot analysis was performed using the antibodies described above. In brief, total cell lysates were separated by SDS-PAGE (5–20% gels; ATTO, Tokyo, Japan) under reducing conditions and then transferred to a PVDF membrane (PALL, SP, Brazil). Antibody reactions were performed with Can Get Signal^®^ Immunoreaction Enhancer Solution (Toyobo). Peroxidase activities on the membrane were visualized with ECL Western Blotting Detection Reagents (GE Healthcare) or ImmunoStar^®^ LD (Wako Pure Chemical Industries, Osaka, Japan), and a ChemiDoc^TM^ MP Imaging System (Bio-Rad, Hercules, CA, USA).

### Reverse transcription and quantitative PCR analysis

Total RNA of the cells was isolated using Sepasol-RNA I Super G (Nacalai Tesque), and then subjected to reverse transcription with Prime Script RTase (Takara Bio). Realtime PCR was performed with FastStart Universal SYBR Green Master (Rox) (Roche Diagnostics, Basel, Switzerland) using StepOnePlus Real-Time PCR System (Applied Biosystems, Life technologies). The nucleotide sequences of the primers are as follows: the forward primer for DYRK1A, 5′-GACCAAAGATGGAAAACGGGA-3′; the reverse primer for DYRK1A, 5′-CCTCCTGTTTCCACTCCAAGAA-3′; the forward primer for GAPDH, 5′-ACGGATTTGGTCGTATTGGG-3′; and the reverse primer for GAPDH, 5′-GTAGTTGAGGTCAATGAAGGGGTC-3′.

### ELISA of the CDC37-nanoKAZ interaction

Stable cell lines transfected with expression vectors for 3xFLAG-tagged DYRK family kinases and its mutants were lysed in ice-cold HENG buffer (50 mM HEPES-KOH, pH 7.9, 150 mM NaCl, 20 mM Na_2_MoO_4_, 2 mM EDTA, 5% Glycerol, 0.5% TritonX-100) containing protease inhibitor cocktail (Nacalai Tesque) on ice. The cleared lysates were added to 96-well plates (OptiPlate-96 HB; PerkinElmer, Waltham, MA, USA) coated with antibody against FLAG peptide (clone M2) diluted in sodium bicarbonate buffer (pH 9.6), and incubated at 4 °C for 3 h. The wells were wash three times with ice-cold HENG buffer and then a luminescence assay was performed.

The luminescence activity of the cell lysates was determined using a Centro LB 960 Microplate Luminometer (Berthold Technologies, Bad Wildbad, Germany). A reaction mixture containing *bis*-coelenterazine (final concentration of 0.1–1 μg/100 μL) in PBS containing 0.02% Tween20 and 20 mM EDTA was injected. The luminescence intensity was recorded at 0.1 s intervals for 10 s or 10 min. The maximum luminescence intensity (*I*_max_), represented in relative luminescence units (rlu), was used in the study.

After measuring the luminescence intensities, the wells were washed three times with HENG buffer, and incubated with HRP-conjugated anti-FLAG antibody diluted with PBS containing 5% Tween20 and 2% fetal bovine serum at 37 °C for one hour. After extensive washing, TMD Super Sensitive One Component HRP Microwell Substrate (SurModics, Eden Prairie, MN, USA) was added into the well and the reaction was stopped with sulfuric acid. Absorbance at 450 nm was measured with ARVO X5 (PerkinElmer). The relative amount of protein bound on the well was estimated from the absorbance.

In Supporting Figure S1a, the values of *I*_max_ are shown in kinetics plots. In Figs 2c,e, 3c, S1d, S3, and S4, the relative luminescence intensities are shown as the fold-change in the *I*_max_ values of the samples relative to *I*_max_ with EGFP. In Figs 4, 5a,c and 6c, the relative luminescence intensities were normalized to the amount of the bound 3xFLAG-DYRK1A mutant and calculated as the fold-change relative to the value at 0 μM of the compounds. Nonspecific binding of CDC37-nanoKAZ to the well did not need to be taken into account because the background luminescence caused by nonspecific binding (EGFP sample) was small compared with the luminescence intensities of the 3xFLAG-DYRK family kinases and its mutants (Figs 2c,e, 3c and S1d).

Small molecules in our original synthetic chemical library, including unpublished small molecule inhibitors of DYRK1A and its structural derivatives (data not shown), were added to the 96-well plates containing the transfected cells at a final concentration of 4 μM. The cells were then processed as described above.

### *In vitro* kinase assay

Detailed information on the assay conditions is available on the website of Carna Biosciences (http://www.carnabio.com/english/index.html; Kobe, Japan). In brief, full-length human recombinant kinases were expressed using a baculovirus expression system as amino-terminal GST-fusion proteins, and purified by glutathione-Sepharose chromatography. The GenBank accession numbers of DYRK1A, DYRK1B, DYRK2, and DYRK3 were NP_001387.2, NP_004705.1, NP_003574.1, and NP_003573.2. Kinase activities were evaluated by Off-chip Mobility Shift Assay after reaction of 1 μM of the substrate DYRKtide-F with ATP (25 μM for DYRK1A, 50 μM for DYRK1B, 10 μM for DYRK2, and 5 μM for DYRK3). For each compound, a DMSO solution was diluted in assay buffer to yield a final concentration of 1% DMSO. After incubation for one hour at room temperature, substrate phosphorylation was analyzed by electrophoretic separation of the substrate and products using a Caliper LC3000 platform (Caliper Life Sciences, Mountain View, CA, USA). The phosphorylated product (P)/(P + substrate) ratio was calculated at each concentration of each small molecule and the percent inhibition was expressed relative to a control assay in the absence of the small molecule. Staurosporine was used as a reference inhibitor in each kinase assay. The IC_50_ of each compound was calculated by interpolation on a log-concentration-response curve fitted with a four-parameter logistic equation.

The effects of 1 μM CaNDY against 275 kinases (listed in Supporting Table S1) were tested using the QuickScout screening assist Mobility Shift Assay or with an immobilized metal ion affinity-based fluorescence polarization screening express kit with an ATP concentration at the *K*_*m*_ or 1 mM. All kinase assays were carried out at Carna Biosciences. The inhibitory map was made with Kinome Render^36^.

### ATP kinetics assay

The kinase reaction for ATP kinetics was performed in a reaction mixture containing serially diluted inhibitors, 10 mM MOPS-KOH (pH 7.0), 10 mM magnesium acetate, 200 μM EDTA, 2.5–40 μM ATP, 0.125–0.4 μCi [γ-^32^P]ATP, 5 μM DYRKtide (Anaspec, CA, USA), and 1 nM of GST-tagged human DYRK1A (Cat# PV3785, Life Technologies), for a final volume of 25 μl. The final concentration of DMSO was adjusted to 0.1%, regardless of the inhibitor concentration. The reaction mixture was incubated at 30 °C for 15 min, and phosphoric acid (final 5%) was then added to stop the reaction. 25 μL of the reaction mixture was dispensed onto P81, a phosphocellulose membrane (Whatman, GE Healthcare), and washed four times in 5% phosphoric acid. Cherenkov light from the incorporated ^32^P was measured using a liquid scintillation counter. The kinase assay conditions, including the incubation period and the concentration of the kinases and substrates, were optimized to maintain linearity during the incubation. The net radioactivity was determined by subtracting the background count from the reaction mixture without kinase. The amount of incorporated ^32^P was calculated from the standard line. The *K*_m_ and *K*_i_ values were calculated with Prism6 software (GraphPad Software, San Diego, CA, USA) using the competitive inhibition model.

### Statistical analysis

Statistical analysis of experimental data was performed by Mann-Whitney test. Results are shown as means ± SD with p values (*p < 0.05, **p < 0.01, ***p < 0.001). Data were fitted to a four-parameter logistic curve (variable slope) for Hill slope determination, from which IC_50_ and EC_50_ values were calculated, using Prism 6.0.

## Additional Information

**How to cite this article**: Sonamoto, R. *et al.* Identification of a DYRK1A Inhibitor that Induces Degradation of the Target Kinase using Co-chaperone CDC37 fused with Luciferase nanoKAZ. *Sci. Rep.* **5**, 12728; doi: 10.1038/srep12728 (2015).

## Supplementary Material

Supporting Information
