# Supplementary material for: Identification of a DYRK1A Inhibitor that Induces Degradation of the Target Kinase using Co-chaperone CDC37 fused with Luciferase nanoKAZ
Source: Sci Rep. 2015 Aug 3;5:12728. doi: 10.1038/srep12728 (PMC4522657; doi:10.1038/srep12728)
Supplement: Supporting Information [file srep12728-s1.pdf]

Supporting Information

Identification of a DYRK1A Inhibitor that Induces Degradation of the Target Kinase using Co-chaperone CDC37 fused with Luciferase nanoKAZ

Rie Sonamoto[1,2], Isao Kii[1,5,*], Yuka Koike[1,5], Yuto Sumida[3,6], Tomoe Kato-Sumida[3,6], Yukiko Okuno[4], Takamitsu Hosoya[3], and Masatoshi Hagiwara[1,*]

*Correspondence: hagiwara.masatoshi.8c@kyoto-u.ac.jp, isao.kii@riken.jp

[1]Department of Anatomy and Developmental Biology, Graduate School of Medicine, Kyoto University, Yoshida-Konoe-cho, Sakyo-ku, Kyoto 606-8501, Japan

[2]Laboratory of Functional Biology, Graduate School of Biostudies, Kyoto University, Yoshida-Konoe-cho, Sakyo-ku, Kyoto 606-8501, Japan

[3]Laboratory of Chemical Bioscience, Institute of Biomaterials and Bioengineering, Tokyo Medical and Dental University, 2-3-10 Kanda-Surugadai, Chiyoda-ku, Tokyo 101-0062, Japan

[4]Medical Research Support Center, Graduate School of Medicine, Kyoto University, Yoshida-Konoe-cho, Sakyo-ku, Kyoto 606-8501, Japan

[5]Present Address: Pathophysiological and Health Science Team, Imaging Application Group, Division of Bio-Function Dynamics Imaging, RIKEN Center for Life Science Technologies, 6-7-3 Minatojima-minamimachi, Chuo-ku, Kobe, Hyogo 650-0047, Japan

[6]Present Address: Chemical Biology Team, Imaging Chemistry Group, Division of Bio-Function Dynamics Imaging, RIKEN Center for Life Science Technologies, 6-7-3 Minatojima-minamimachi, Chuo-ku, Kobe, Hyogo 650-0047, Japan

***Contact information**

Masatoshi Hagiwara, MD and PhD

Department of Anatomy and Developmental Biology, Graduate School of Medicine, Kyoto

University, Yoshida-Konoe-cho, Sakyo-ku, Kyoto 606-8501, Japan

E-mail: hagiwara.masatoshi.8c@kyoto-u.ac.jp

Tel: +81-75-753-4341 and Fax: +81-75-751-7529

Isao Kii, PhD

Pathophysiological and Health Science Team, Imaging Application Group, Division of Bio-Function Dynamics Imaging, RIKEN Center for Life Science Technologies, 6-7-3 Minatojima-minamimachi, Chuo-ku, Kobe, Hyogo 650-0047, Japan

E-mail: isao.kii@riken.jp

Tel: +81-78-304-7124 (Ex. 95-8386) and Fax: 078-304-7126

Supporting Figures

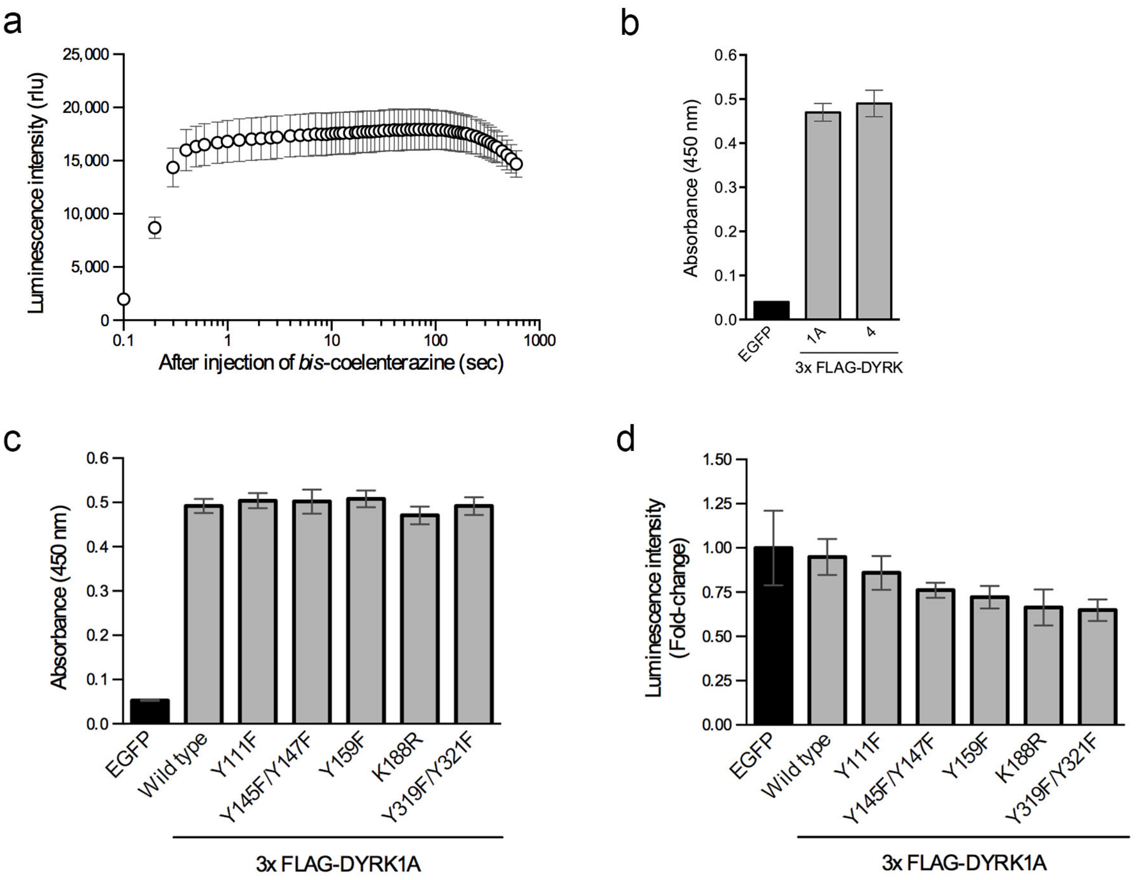

Figure S1. Development of a bioluminescent co-chaperone CDC37.

(a) Luminescence kinetics of total cell lysate (about 7 ng diluted in 5 μL of HENG buffer) from transfected 293T cells. Points are means ± SD (n = 5).

(b) Amounts of 3xFLAG-DYRK1A and 3xFLAG-DYRK4 bound on an antibody-coated well, as used in Figure 2c. Bound proteins were quantified with HRP-conjugated antibody against FLAG. Absorbance values are shown. Amounts of bound 3xFLAG-DYRK1A proteins were almost the same between the samples (within ± 2.0%). Bar graphs show means ± SD (n = 8). (c) Amounts of 3xFLAG-DYRK1A and its mutants bound on an antibody-coated well, as used in Figure 2e. Bound proteins were quantified, and absorbance values are shown. Amounts of bound 3xFLAG-DYRK1A proteins were almost the same between the samples (within ± 3.5%). Bar graphs show means ± SD (n = 5). (d) DYRK1A proteins did not interact with nanoKAZ. The luminescence intensities of the complexes between nanoKAZ and the 3xFLAG-tagged DYRK1A

proteins bound on a 96-well plate coated with antibody against FLAG are shown as fold-changes relative to that with EGFP. Bar graphs show means ± SD (n = 5).

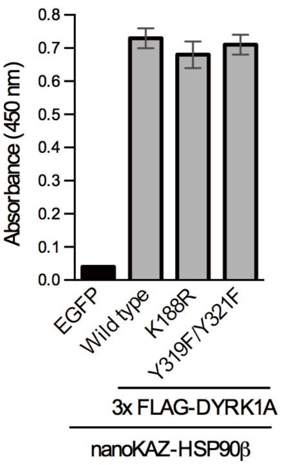

Figure S2. Development of a bioluminescent chaperone HSP90β.

Amounts of 3xFLAG-DYRK1A proteins bound on an antibody-coated well, as used in Figure 3c. Bound proteins were quantified with HRP-conjugated antibody against FLAG. Absorbance values are shown. Amounts of bound 3xFLAG-DYRK1A proteins were almost the same between the samples (within ± 4.0%). Bar graphs show means ± SD (n = 4).

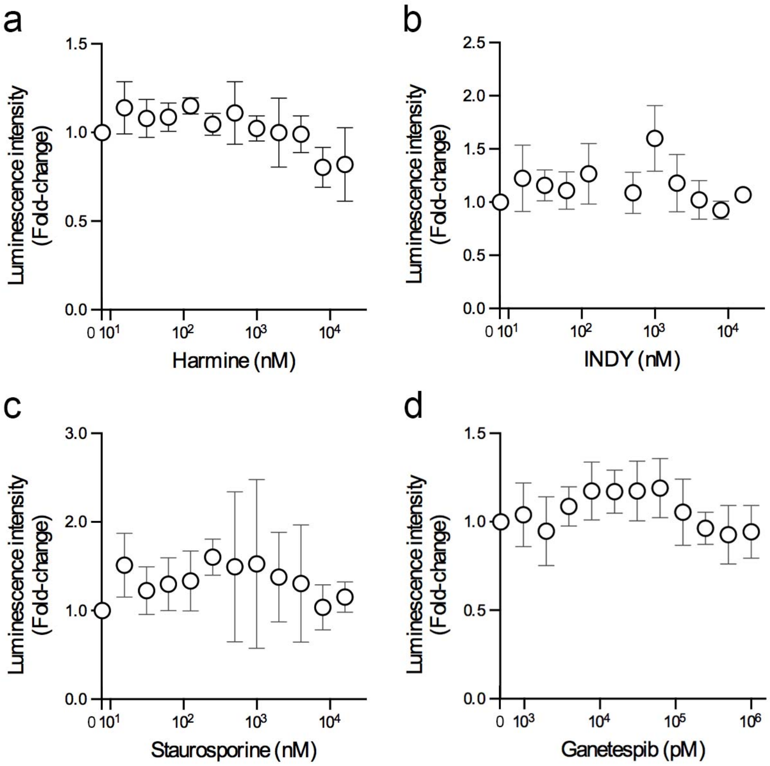

Figure S3. DYRK1A inhibitors and a HSP90 inhibitor did not inhibit the luminescent activity of CDC37-nanoKAZ.

(a-d) Luminescence intensities of total cell lysates of cells treated with the indicated concentrations of harmine (a), INDY (b), staurosporine (c), and ganetespib (d), as also used in Figure 4. Luminescence intensities at each concentration are shown as fold-changes relative to that at 0 nM. Points are means ± SD (n = 3).

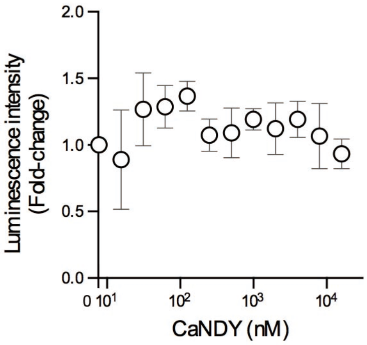

Figure S4. CaNDY did not inhibit the luminescent activity of CDC37-nanoKAZ.
Luminescence intensities of total cell lysates of cells treated with the indicated concentrations of CaNDY, as also used in Figure 5c. Luminescence intensities at each concentration are shown as fold-changes to that at 0 nM. Points are means ± SD (n = 3).

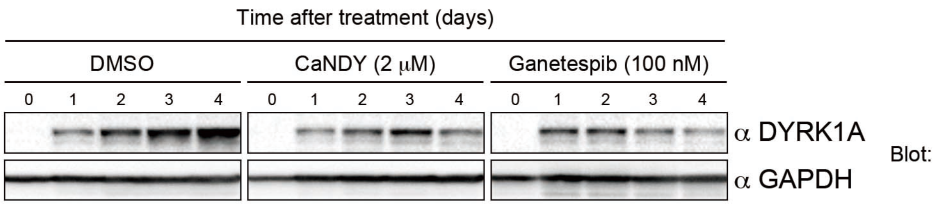

Figure S5. CaNDY and ganetespib prevented accumulation of endogenous DYRK1A. HEK293 cells were cultured in the presence of CaNDY (2 μM) or ganetespib (100 nM) for four days. Total cell lysates were collected at the indicated time point (day 0-4), and subjected to SDS-PAGE followed by Western blot analysis.

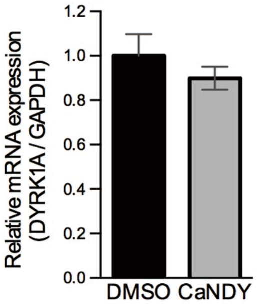

Figure S6. Expression of endogenous DYRK1A mRNA in HEK293 cells treated with CaNDY.

HEK293 cells were cultured in the presence of CaNDY (2 μM) for four days, and subjected to reverse transcription followed by quantitative PCR analysis. Bar graphs show means ± SD (n = 3).

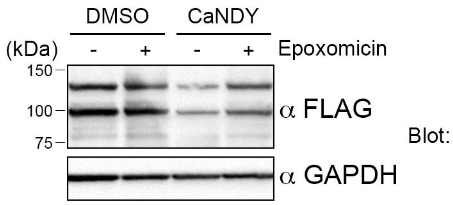

Figure S7. Proteasome inhibitor epoxomicin partially prevented the CaNDY-mediated degradation of 3xFLAG-DYRK1A.

293T cells were transiently transfected with an expression vector for 3xFLAG-DYRK1A. At 24 h after transfection, the cells were pre-treated with 2 μM epoxomicin for 1 h to inhibit proteasome irreversibly, and then treated with/without CaNDY (2 μM) for 8 h. The total cell lysates were subjected to SDS-PAGE followed by Western blot analysis.

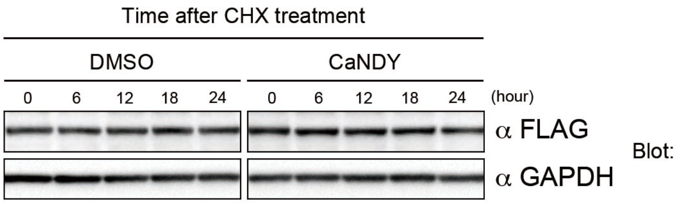

Figure S8. CaNDY did not destabilize pre-accumulated DYRK1A in HEK293 cells. HEK293 cells were transfected with an expression vector for 3xFLAG-DYRK1A. At 48 h after transfection, the cells were pre-treated with 1 mg/mL cycloheximide (CHX) to stop protein synthesis, and then treated with CaNDY (2 μM) for the indicated time (0-24 h). The total cell lysates were subjected to SDS-PAGE followed by Western blot analysis.

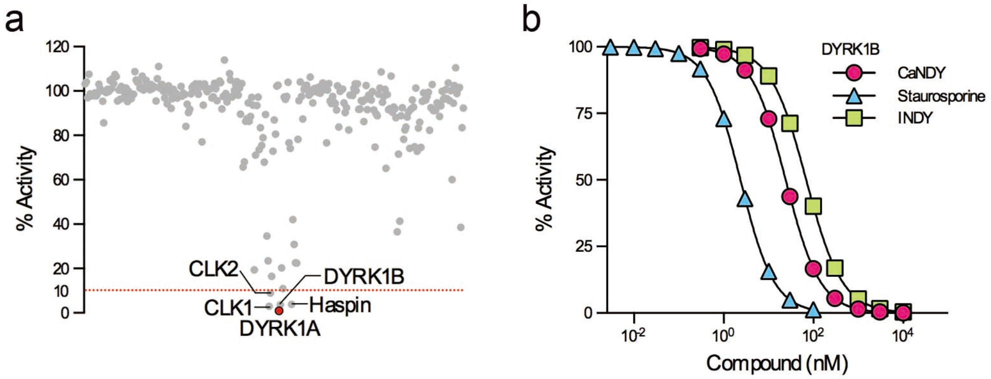

Figure S9. CaNDY inhibited the *in vitro* kinase activity of DYRK family kinases.

(a) Inhibitory activities of CaNDY (1 μM) against 275 recombinant kinases. The red point indicates DYRK1A. The red dashed line indicates 10% of the kinase activity. The inhibitory activities are shown in Table S1. (b) Recombinant DYRK1B was incubated with the substrate peptide DYRKtide-F in the presence of different concentrations of CaNDY, INDY, and staurosporine. CaNDY, INDY, and staurosporine inhibited the kinase activity with IC_{50} values of 24.1 nM, 69.1 nM, and 2.36 nM, respectively. Representative dose-response curves with Hill slopes are shown.

Supporting Tables

Table S1.

Average of two replicates is shown as percent activity of kinase activity in the presence of CaNDY (1 μM) relative to solvent control (DMSO)

DYRK1A, DYRK1B, CLK1, CLK2, and Haspin were inhibited by over 90%.

Kinase	%Activity	Kinase	%Activity
ABL	103.2	ABL(E255K)	104.1
ABL(T315I)	97.0	ACK	107.6
ALK	98.7	ALK(F1174L)	97.9
ALK(L1196M)	97.8	ALK(R1275Q)	98.5
EML4-ALK	99.2	NPM1-ALK	97.8
ARG	98.8	AXL	103.1
BLK	101.0	BMX	100.1
BRK	95.4	BTK	85.7
CSK	99.7	DDR1	99.3
DDR2	100.8	EGFR	99.9
EGFR(d746-750)	104.8	EGFR(d746-750/T790M)	101.0
EGFR(L858R)	105.7	EGFR(L861Q)	103.3
EGFR(T790M)	107.5	EGFR(T790M/L858R)	106.6
EPHA1	95.5	EPHA2	100.5
EPHA3	96.3	EPHA4	102.1
EPHA5	100.1	EPHA6	96.8
EPHA7	97.6	EPHA8	101.3
EPHB1	95.3	EPHB2	99.8
EPHB3	98.6	EPHB4	96.6
FAK	98.5	FER	98.6
FES	108.7	FGFR1	104.9
FGFR2	103.9	FGFR3	104.0
FGFR3(K650E)	101.4	FGFR3(K650M)	101.0
FGFR4	106.7	FGFR4(N535K)	100.5

FGFR4(V550E)	102.1	FGFR4(V550L)	104.8
FGR	101.8	FLT1	102.0
FLT3	100.6	FLT4	102.9
FMS	101.0	FRK	102.1
FYN	101.7	HCK	95.5
HER2	105.5	HER4	111.0
IGF1R	101.3	INSR	102.8
IRR	103.0	ITK	99.9
JAK1	96.9	JAK2	100.3
JAK3	94.2	KDR	102.3
KIT	99.3	KIT(D816V)	92.2
KIT(T670I)	96.6	KIT(V560G)	100.8
KIT(V654A)	98.5	LCK	99.0
LTK	96.9	LYNa	97.6
LYNb	98.0	MER	102.0
MET	97.7	MET(Y1235D)	99.0
MUSK	98.4	PDGFRα	84.1
PDGFRα(T674I)	94.0	PDGFRα(V561D)	90.3
PDGFRβ	91.7	PYK2	98.7
RET	101.6	RET(G691S)	102.4
RET(M918T)	102.1	RET(S891A)	103.2
RET(Y791F)	100.2	RON	98.6
ROS	103.2	SRC	102.1
SRM	77.0	SYK	93.7
TEC	103.2	TIE2	101.0
TNK1	99.7	TRKA	100.9
TRKB	100.9	TRKC	101.0
TXK	96.8	TYK2	97.4
TYRO3	103.5	YES	101.1
ZAP70	95.7	AKT1	103.7
AKT2	104.6	AKT3	103.8

AMPKα1/β1/γ1	91.9	AMPKα2/β1/γ1	97.5
AurA	113.9	AurA/TPX2	98.8
AurB	95.1	AurC	97.0
BRAF_Cascade	107.9	BRAF(V600E)_Cascade	104.9
BRSK1	97.3	BRSK2	97.8
CaMK1α	100.3	CaMK1δ	103.0
CaMK2α	104.2	CaMK2β	98.2
CaMK2γ	101.4	CaMK2δ	89.6
CaMK4	103.4	CDC2/CycB1	65.7
CDC7/ASK	68.0	CDK2/CycA2	86.3
CDK2/CycE1	87.5	CDK3/CycE1	94.7
CDK4/CycD3	86.4	CDK5/p25	88.5
CDK6/CycD3	97.1	CDK7/CycH/MAT1	93.6
CDK9/CycT1	19.4	CGK2	71.1
CHK1	93.5	CHK2	97.2
CK1α	73.6	CK1γ1	83.4
CK1γ2	75.4	CK1γ3	79.0
CK1δ	67.9	CK1ε	89.4
CK2α1/β	34.7	CK2α2/β	23.4
CLK1	3.0	CLK2	9.0
CLK3	16.5	COT_Cascade	72.9
CRIK	87.0	DAPK1	80.6
DCAMKL2	102.2	DLK_Cascade	102.6
DYRK1A	0.9	DYRK1B	3.7
DYRK2	20.3	DYRK3	11.0
EEF2K	97.2	Erk1	96.2
Erk2	97.6	Erk5	103.9
GSK3α	76.6	GSK3β	74.1
Haspin	3.8	HGK	42.1
HIPK1	30.9	HIPK2	22.6
HIPK3	22.4	HIPK4	78.2

IKKα	95.7	IKKβ	104.1
IKKε	97.6	IRAK1	87.4
IRAK4	100.8	JNK1	102.4
JNK2	102.6	JNK3	100.8
LATS2	98.1	LOK	106.3
MAP2K1_Cascade	99.3	MAP2K2_Cascade	94.7
MAP2K3_Cascade	101.1	MAP2K4_Cascade	100.3
MAP2K5_Cascade	101.4	MAP2K6_Cascade	99.5
MAP2K7_Cascade	101.7	MAP3K1_Cascade	99.0
MAP3K2_Cascade	101.6	MAP3K3_Cascade	103.7
MAP3K4_Cascade	100.6	MAP3K5_Cascade	102.6
MAP4K2	76.7	MAPKAPK2	100.5
MAPKAPK3	103.4	MAPKAPK5	95.5
MARK1	94.4	MARK2	97.5
MARK3	95.0	MARK4	94.3
MELK	91.5	MGC42105	102.3
MINK	83.7	MLK1_Cascade	99.9
MLK2_Cascade	123.6	MLK3_Cascade	100.9
MNK1	94.6	MNK2	90.3
MOS_Cascade	92.4	MRCKα	128.5
MRCKβ	82.5	MSK1	98.1
MSK2	98.3	MSSK1	98.0
MST1	101.2	MST2	103.2
MST3	100.2	MST4	103.2
NDR1	91.8	NDR2	91.9
NEK1	95.6	NEK2	95.3
NEK4	86.2	NEK6	96.1
NEK7	102.5	NEK9	91.6
NuaK1	98.1	NuaK2	98.1
p38α	95.9	p38β	99.7
p38γ	91.4	p38δ	74.8

p70S6K	89.7	p70S6Kβ	88.9
PAK1	98.6	PAK2	101.3
PAK3	108.6	PAK4	95.0
PAK5	97.5	PAK6	100.7
PASK	65.1	PBK	98.4
PDHK2	91.6	PDHK4	92.0
PDK1	106.7	PEK	90.4
PGK	92.8	PHKG1	92.1
PHKG2	88.0	PIM1	36.5
PIM2	71.6	PIM3	41.4
PKACα	88.9	PKACβ	92.5
PKACγ	78.2	PKCα	82.4
PKCβ1	83.8	PKCβ2	88.2
PKCγ	90.6	PKCδ	74.1
PKCε	91.9	PKCζ	95.9
PKCη	92.8	PKCθ	74.9
PKCι	101.3	PKD1	72.9
PKD2	83.3	PKD3	85.9
PKN1	74.6	PKR	95.4
PLK1	99.2	PLK2	95.9
PLK3	95.9	PRKX	97.6
QIK	99.5	RAF1_Cascade	82.3
ROCK1	92.5	ROCK2	102.9
RSK1	91.2	RSK2	91.1
RSK3	91.4	RSK4	94.6
SGK	93.6	SGK2	88.6
SGK3	93.3	SIK	99.9
skMLCK	96.2	SLK	96.9
SRPK1	100.6	SRPK2	96.6
TAK1-TAB1_Cascade	103.7	TAOK2	95.7
TBK1	100.2	TNIK	60.0

TSSK1	94.5	TSSK2	102.5
TSSK3	110.5	WNK1	98.8
WNK2	100.9	WNK3	101.6
PIK3CA/PIK3R1	38.5	SPHK1	83.5
SPHK2	92.3		

Table S2.

The commercially available antibodies used in this study.

Antibody		Clone ID	Vendor	Cat. No.
ANTI-FLAG M2 antibody	Mouse monoclonal	M2	SIGMA	F-3165
ANTI-FLAG M2-Peroxidase (HRP) antibody	Mouse monoclonal	M2	SIGMA	A8592
GAPDH	Mouse monoclonal	6C5	Abcam	ab8245
CLK1	Rabbit polyclonal		Abcam	ab74044
Haspin	Rabbit polyclonal		Abcam	ab115800
SRPK1	Mouse monoclonal	12/SRPK1	BD Transduction Laboratories	611072
SRPK2	Mouse monoclonal	23/SRPK2	BD Transduction Laboratories	611118
CDK9	Mouse monoclonal	D-7	Santa Cruz Biotechnology	sc-13130
CDC37	Rabbit monoclonal	D11A3	Cell Signaling Technology	4793
DYRK1A	Rabbit polyclonal		Cell Signaling Technology	2771
DYRK1B	Rabbit polyclonal		Cell Signaling Technology	2703
CK2a	Rabbit polyclonal		Cell Signaling Technology	2656
GSK3b	Rabbit monoclonal	27C10	Cell Signaling Technology	9315
ERK1/2 (p42/p44 MAPK)	Rabbit polyclonal		Cell Signaling Technology	9102
p38 MAPK	Rabbit polyclonal		Cell Signaling Technology	9212
SAPK/JNK (JNK2)	Rabbit monoclonal	56G8	Cell Signaling Technology	9258
p70S6K	Rabbit polyclonal		Cell Signaling Technology	9202
AKT1	Rabbit monoclonal	C73H10	Cell Signaling Technology	2938
CHK2	Rabbit polyclonal		Cell Signaling Technology	2662
MARK3	Rabbit polyclonal		Cell Signaling Technology	9311
CK1	Rabbit polyclonal		Cell Signaling Technology	2655
MEK1	Rabbit monoclonal	30C8	Cell Signaling Technology	9146
c-Raf (RAF-1)	Rabbit polyclonal		Cell Signaling Technology	9422
SRC	Rabbit monoclonal	32G6	Cell Signaling Technology	2123

FAK	Rabbit polyclonal		Cell Signaling Technology	3285
FYN	Rabbit polyclonal		Cell Signaling Technology	4023
c-ABL	Rabbit polyclonal		Cell Signaling Technology	2862
IKKa	Rabbit polyclonal		Cell Signaling Technology	2682

Synthesis of small molecules

General remarks: Melting points (mp) were measured on an OptiMelt MPA100 automated melting point apparatus (Stanford Research Systems) and are uncorrected. IR spectra were measured by diffuse reflectance method or single reflection ATR method on a Shimadzu IRPrestige-21 spectrometer attached with DRS-8000A or MIRacle^{TM}A (ZnSe) single reflection ATR accessory with the absorption band given in cm^{-1}. ^{1}H and ^{13}C NMR spectra were measured by JEOL JNM-ECS 400 spectrometer at 400 and 100 MHz. DMSO-d_6 (Cambridge Isotope Laboratories, Cat. No. DLM-10) were used as solvents for obtaining NMR spectra. Chemical shifts (δ) are given in parts per million (ppm) downfield from the solvent peak (δ 2.49 for ^{1}H NMR and δ 39.5 for ^{13}C NMR in DMSO-d_6) as an internal reference with coupling constants (*J*) in hertz (Hz). The abbreviations s, d, t and br signify singlet, doublet, triplet, and broad, respectively.

Synthesis of CaNDY

(*Z*)-5-[(2,3-dihydrobenzofuran-5-yl)methylene]-2-iminothiazolidin-4-one (CaNDY)

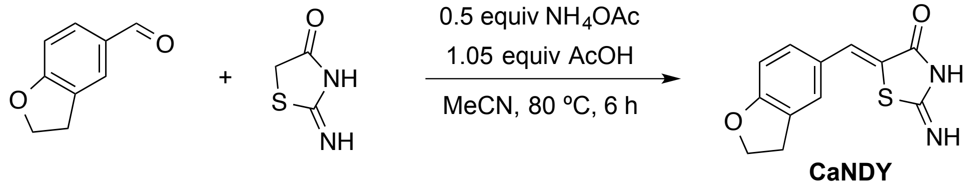

Under an argon atomosphere, to a solution of 2,3-dihydrobenzofuran-5-carbaldehyde (296 mg, 2.00 mmol), pseudothiohydantoin (232 mg, 2.00 mmol), and ammonium acetate (77.0 mg, 0.999 mmol) in acetonitirile (2.0 mL) was added acetic acid (120 µL, 2.10 mmol) at room temperature and the mixture was stirred with heating at 80 ºC (bath temperature) for 6 h. After cooling the reaction mixture to room temperature, the precipitate was collected by filtration using a funnel. The collected solid was washed on the funnel with water (3 mL × 4) and diethyl ether (3 mL × 2), and then dried under reduced pressure to afford (*Z*)-5-[(2,3-dihydrobenzofuran-5-yl)methylene]-2-iminothiazolidin-4-one (CaNDY) (492 mg,

quantitative) as a pale yellow solid.

mp 280 ºC (dec); IR (KBr, cm^{-1}) 3177, 3049, 3028, 2951, 2922, 1667, 1603, 1584, 1497, 1427, 1302, 1271, 1233, 1204, 1150, 760; ^{1}H NMR (400 MHz, DMSO-d_6) δ 9.28 (br s, 1H), 9.07 (br s, 1H), 7.52 (s, 1H), 7.43 (d, J = 1.2 Hz, 1H), 7.34 (dd, J = 8.4, 1.2 Hz, 1H), 6.89 (d, J = 8.4 Hz, 1H), 4.59 (t, J = 8.8 Hz, 2H), 3.23 (t, J = 8.8 Hz, 2H); ^{13}C NMR (100 MHz, DMSO-d_6) δ 180.6, 175.4, 161.1, 130.6, 129.5, 128.8, 126.5, 126.2, 125.7, 109.7, 71.7, 28.7.

(*Z*)-5-[(3,4-methylenedioxyphenyl)methylene]-2-iminothiazolidin-4-one (PD0439)

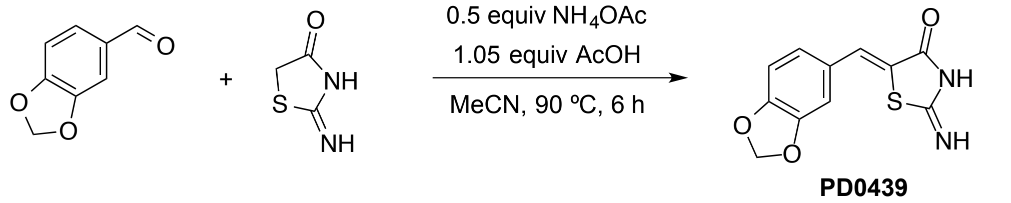

Under an argon atomosphere, to a solution of piperonal (300 mg, 2.00 mmol), pseudothiohydantoin (232 mg, 2.00 mmol), and ammonium acetate (77.0 mg, 0.999 mmol) in acetonitirile (2.0 mL) was added acetic acid (120 μL, 2.10 mmol) at room temperature and the mixture was stirred with heating at 90 ºC (bath temperature) for 6 h. After cooling the reaction mixture to room temperature, the precipitate was collected by filtration using a funnel. The collected solid was washed on the funnel with water (3 mL × 4) and diethyl ether (3 mL × 2), and then dried under reduced pressure to afford (*Z*)-5-[(3,4-methylenedioxyphenyl)methylene]-2-iminothiazolidin-4-one (295 mg, 59.5%) as a pale yellow solid.

mp 300 ºC (dec); IR (cm^{-1}) 3206, 2926, 1665, 1591, 1485, 1443, 1368, 1354, 1236, 1146, 1036, 924, 758; ^{1}H NMR (400 MHz, DMSO-d_6) δ 9.41 (br s, 1H), 9.15 (br s, 1H), 7.56 (s, 1H), 7.18–7.09 (m, 3H), 6.15 (s, 2H); ^{13}C NMR (100 MHz, DMSO-d_6) δ 217.9, 212.8, 185.9, 185.5, 166.6, 165.8, 164.6, 162.2, 146.5, 146.2, 139.3.

(*Z*)-5-[(1*H*-indol-5-yl)methylene]-2-thioxothiazolidin-4-one (RD0440)

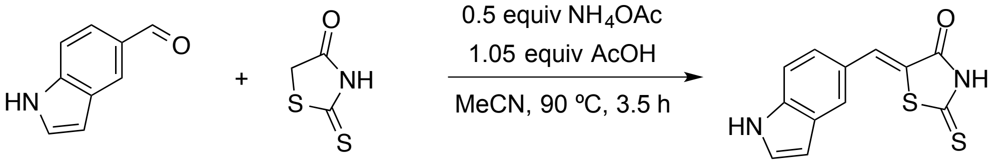

RD0440

Under an argon atomosphere, to a solution of indole-5-carboxaldehyde (290 mg, 2.00 mmol), rhodanine (266 mg, 2.00 mmol), and ammonium acetate (77.0 mg, 0.999 mmol) in acetonitirile (2.0 mL) was added acetic acid (120 µL, 2.10 mmol) at room temperature and the mixture was stirred with heating at 90 ºC (bath temperature) for 3.5 h. After cooling the reaction mixture to room temperature, the precipitate was collected by filtration using a funnel. The collected solid was washed on the funnel with water (3 mL × 4) and diethyl ether (3 mL × 2), and then dried under reduced pressure to afford (*Z*)-5-[(1*H*-indol-5-yl)methylene]-2-thioxothiazolidin-4-one (426 mg, 81.8%) as an orange solid.

mp 285 ºC (dec); IR (cm^{-1}) 3387, 1682, 1584, 1568, 1445, 1422, 1236, 1200, 1157, 1125, 1065, 791, 764, 723, 685, 654; ^{1}H NMR (400 MHz, DMSO-d_6) δ 11.56 (br s, 1H), 7.92 (s, 1H), 7.80 (s, 1H), 7.59 (d, J = 8.8 Hz, 1H), 7.51 (d, J = 2.8 Hz, 1H), 7.38 (d, J = 8.8 Hz, 1H), 6.64 (d, J = 2.8 Hz, 1H) (the signal for the NH of thioxothiazolidinone was not clearly observed); ^{13}C NMR (100 MHz, DMSO-d_6) δ 233.3, 207.0, 174.6, 172.1, 165.8, 165.1, 162.1, 161.5, 161.1, 158.2, 150.1, 140.1.

(*Z*)-5-[(1*H*-indol-5-yl)methylene]-2-iminothiazolidin-4-one (PD0442)

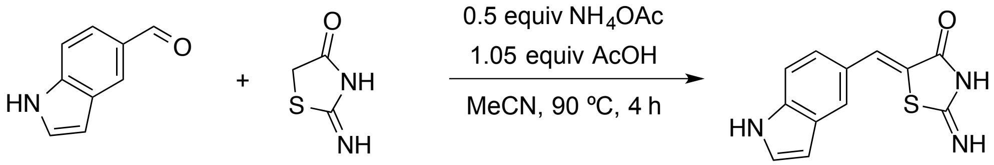

PD0442

Under an argon atomosphere, to a solution of indole-5-carboxaldehyde (290 mg, 2.00 mmol), pseudothiohydantoin (232 mg, 2.00 mmol), and ammonium acetate (77.0 mg, 0.999 mmol) in acetonitirile (2.0 mL) was added acetic acid (120 µL, 2.10 mmol) at room temperature and the mixture was stirred with heating at 90 ºC (bath temperature) for 4 h. After cooling the reaction

mixture to room temperature, the precipitate was collected by filtration using a funnel. The collected solid was washed on the funnel with water (3 mL × 4) and diethyl ether (3 mL × 2), and then dried under reduced pressure to afford (*Z*)-5-[(1*H*-indol-5-yl)methylene]-2-iminothiazolidin-4-one (382 mg, 64.2%) as a dark purple solid.

mp 315 ºC (dec); IR (cm^{-1}) 3184, 3021, 1670, 1645, 1593, 1493, 1454, 1416, 1396, 1366, 1348, 1248, 1213, 1128, 764, 723, 654; ^{1}H NMR (400 MHz, DMSO-d_6) δ 11.44 (br s, 1H), 7.84 (s, 1H), 7.73 (s, 1H), 7.56 (d, *J* = 8.4 Hz, 1H), 7.48 (d, *J* = 2.8 Hz, 1H), 7.36 (d, *J* = 8.4 Hz, 1H), 6.58 (d, *J* = 2.8 Hz, 1H) (the signals for two NH of iminothiazolidinone were not clearly observed); ^{13}C NMR (100 MHz, DMSO-d_6) δ 218.2, 213.2, 173.9, 168.9, 165.6, 164.5, 162.7, 162.5, 160.3, 159.9, 149.7, 139.5.
